# Polyamidoamine Dendron-Bearing Lipids as Drug-Delivery Excipients

**DOI:** 10.3390/molecules27227817

**Published:** 2022-11-13

**Authors:** Ender Sarigul, Merve Zaim, Mehmet Senel, Tugba Sagir, Sevim Isik

**Affiliations:** 1 BSI Group, Kozyatagi, Istanbul 34742, Turkey; 2 SANKARA Brain and Biotechnology Research Center, Avcilar, Istanbul 34320, Turkey; 3 Department of Biochemistry, Faculty of Pharmacy, Biruni University, Istanbul 34010, Turkey; 4 Pim Grup Cosmetics Consultancy, Gokturk, Istanbul 34077, Turkey; 5 Department of Molecular Biology and Genetics, Faculty of Science and Engineering, Uskudar University, Uskudar, Istanbul 34662, Turkey

**Keywords:** dendritic amphiphiles, drug solubility, drug delivery, polyamidoamine, micelles, cytotoxic activity

## Abstract

An amine-terminated polyamidoamine (PAMAM) dendron and two long alkyl groups were designed as a novel drug carrier that possesses an interior for the encapsulation of drugs and a biocompatible surface. We synthesized three dendron-bearing lipids, DL-G1, DL-G2, and DL-G3, which included first, second, and third generation polyamidoamine dendrons, respectively. The synthesized dendrimer encapsulating anticancer drug, 5-fluorouracil (5-FU), was prepared by extraction with chloroform from mixtures of the dendrimers and varying amounts of the drug. In vitro cytotoxicity of PAMAM conjugated di-n-dodecylamine micelles (G1, G2, G3) were analyzed on human gastric adenocarcinoma cells (AGS) by water-soluble tetrazolium-1 (WST-1) cell proliferation assay. Upon exposure to 5-FU loaded micelles, the viability of the cells decreased gradually in all generations. Cytotoxicity increased with increasing generation and reached its highest rate of 69.8 ± 3.2% upon 15 µM 5FU-loaded 25 µM PAMAM DL-3 micelle treatment. These results demonstrate that 5FU-loaded PAMAM conjugated di-n-dodecylamine treatment inhibits the proliferation of AGS cells in a generation-dependent manner.

## 1. Introduction

Cancer is a leading cause of death worldwide and is responsible for approximately 13% of all deaths, according to the World Health Organization. Chemotherapy is an essential component in the multidisciplinary management of most cancers. The anticancer drug 5-FU is widely used for the treatment of a wide range of cancer types, including colorectal and breast cancers and cancers of the aerodigestive tract [1]. It is a BCS class III drug that shows good water solubility and poor permeability; i.e., it is a hydrophilic drug having 12.20 mg ml^−1^ [2]. Due to the high toxicity of the drug, it cannot generally be used in adequate doses and/or adequate periods of treatment. Therefore, 5-FU started to be used with other chemotherapeutic agents to increase the therapeutic index. Nonetheless, the response rates for 5-FU-based chemotherapy as a first-line treatment for advanced colorectal cancer are only in the range of 10–15% [3]. The combination of 5-FU with newer chemotherapeutics, such as irinotecan and oxaliplatin, has improved the response rates for advanced colorectal cancer to 40–50% [3].

The other major problem with the vast majority of clinically used drugs is their short half-life in the bloodstream and their high overall clearance rate. A relatively small amount of the drug reaches the target site to provide the desired reaction, while the non-selective distribution in the body gives undesired reactions and leads to side effects [4]. For these reasons, the applied drug dose is reduced to minimize these side effects; however, with such a reduction, the full therapeutic potential of the drug is not achieved [4]. To overcome these problems and to increase the therapeutic index, a new drug-delivery concept with polymeric carriers is currently the major topic of research [5]. Solubilization represents one of the major current challenges in drug development, because approximately 40% of the new compounds are poorly water-soluble [6]. The development of new drug formulations is not sufficient by itself; a system is also required to overcome the solubility problem that solubilizers currently fail to overcome [7]. For these purposes, formulations comprising multiple doses of drugs in a single vehicle, such as a natural or synthetic polymer, can triumph where solubilizers fail [7].

Natural and synthetic polymers are always fascinating for scientists in every area of research, but synthetic biodegradable polymers, in particular, are very important for pharmaceutical and biomedical applications, due to their structure control and low toxicity [8]. In this context, dendrimers have emerged as one of the most promising drug carriers for different therapeutic categories and bioactive agents [8]. Dendrimers are promising candidates in overcoming solubility problems that limit drug development [9]. Dendrimers can secrete water-insoluble molecules between their branches and easily carry them in the blood system along the body. The solubility efficiency of dendrimers can be manipulated by simply modifying their cores with different branching units, using a different core with the same branches, modifying the end groups, and micellizing the dendrimers [10].

Because of their unique properties, dendritic structures represent great potential, compared with the other carrier systems, especially in the field of drug delivery. Dendrimers can be monodispersed, which offers researchers the possibility of working with a tool that is well-defined, reproducible, and of scalable size [8]. Dendrimers with uniform and well-defined sizes and shapes are of prominent interest in biomedical applications because of their ability to cross cell membranes, as well as their ability to reduce the risk of premature clearance from the body. Despite their toxicity, dendrimers have been seen as exceptional drug carriers cause of their ability, as intracellular drug-delivery providers, to pass through biological membranes to circulate in the blood system long enough to exert a clinical effect and to target specific structures [11,12]. Ideally, dendrimers are developed to deliver drugs to target tissues to obtain adequate therapeutic efficacy, avoiding toxic effects on the healthy bystander cells, which are associated with the potential organ-specific toxicity of the free drugs [13].

Micelles are composed of lipids that are similar to those present in biological membranes; therefore, they are expected to be biocompatible, biodegradable, practically non-immunogenic, and non-toxic [14]. In addition, they are well suited for the delivery of therapeutic agents, because they usually provide for a sustained release of drug content, slowly and gradually, with an increased overall drug efficiency [15]. Micelles that are made of dendritic polymers are generally unimolecular (i.e., they have only one hydrophilic and hydrophobic layer); due to their nanosize scale, they are readily accepted into cells via endocytosis [16]. A new insight is needed for cancer treatment, and using dendritic micelles as drug vehicles could be the answer [3]. As the solubility problem is overcome, sufficient doses of a cancer drug can be used. In addition, as the drug will not be circulating freely in the bloodstream, systemic toxicity is expected to be reduced [17].

A recent novel nanocarrier approach—i.e., double or multiple water/oil/water (w/o/w) nanoemulsions—is the use of a system that contains dispersions of minute water droplets inside bigger oil droplets, followed by the whole of the water/oil (w/o) being dispersed in an outer surface water-phase with the support of surfactants [18]. Shishu et al. developed a microemulsion-based oral delivery system of 5-FU with the aim of enhancing its oral bioavailability. Microemulsions were prepared employing Tween-20, Span-20, isopropyl myristate/Captex 200 as an oil phase and triple-distilled water as an aqueous phase [19]. In another work, four different concentrations of 5-FU were used to study the impact on the “ghosts” cell wall. 5-FU was then loaded into the bacterial ghosts and the loading capacity and the entrapment efficiency were determined; they were found to be 38.3 ± 0.8 and 76.6 ±0.8, respectively [20]. Solid lipid nanoparticle (SLN) formulae were also utilized for the release of 5-FU inside the colonic medium for local treatment of colon cancer. SLNs were prepared by a double-emulsion-solvent evaporation technique (w/o/w) using triglyceride esters, Dynasan™ 114, or Dynasan™ 118, along with soyalecithin as the lipid part [21]. The 5-FU was also encapsulated into magnetite–zeolite nanocomposite particles in our previous study [22]. The cCytotoxic effects of 5-FU-loaded MZNC on human gastric carcinoma (AGS) cells were determined by real-time cell analysis and colorimetric WST-1 cell viability assay.

The aim of the study was to investigate the micelle formation and toxicity of dendron-bearing lipids with PAMAM G1, G2, and G3 dendrons. For this reason, a novel cationic amphiphilic lipid with a hydrophilic tail and hydrophobic core was initially synthesized. Then, PAMAM dendrimers were converted to micelles with the ability to carry water-insoluble drugs in their hydrophobic core. For use as an anti-cancer agent, 5-FU was loaded into PAMAM dendrons of first to third generation, DL-G1, DL-G2, and DL-G3. Finally, the in vitro cytotoxicity of PAMAM dendrimers in all generations, with or without 5-FU, was analyzed on AGS cells.

## 2. Results and Discussion

### 2.1. Characterization

**Figure 1:**
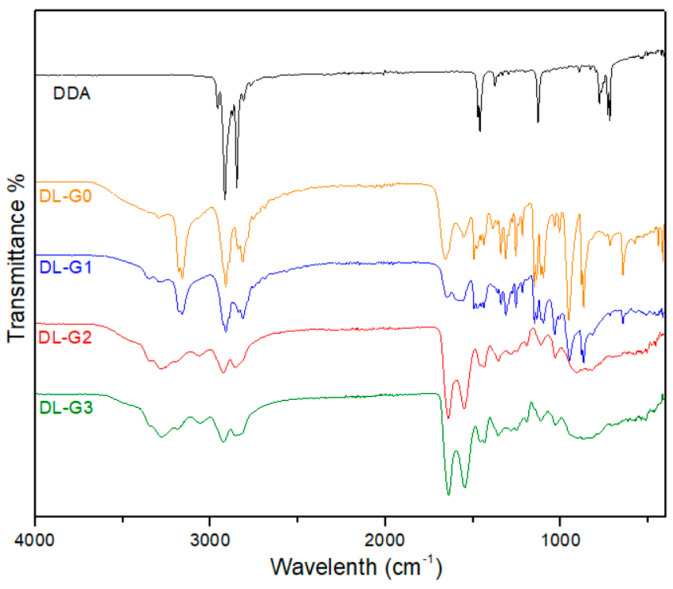
FT-IR analysis of PAMAM dendron-bearing lipids.

In the FT-IR analysis of the dendrimers, several characteristic picks were determined in Figure 1. Because of the nature of the dendrimers, two different reactions were used for building the generations—esterification followed by amination—generally referred to as Michael addition. As only side groups can provide strong peaks in bulky materials such as dendrimers, to confirm those reactions, the signals of the ester and amine groups were checked in every step of the reaction by FT-IR (Figure 1). The FTIR spectra of the full generation dendrimers were of N-H stretch for terminal primary amine at averages of 3330 cm^−1^, 3284 cm^−1^, and 3245 cm^−1^ and C-N stretch of primary amine at averages of 1312 cm^−1^ and 1280 cm^−1^. With increasing generations, the N-H number increased and peaks were seen at higher wavelengths. IR spectra of half generation of dendrimers were of C=O stretch of the carbonyl group; peaks were at an average of 1732 cm^−1^.

#### CMC Analysis

The critical micelle concentration of the new amphiphiles was in range of 2.5 × 10^−6^ to 5.0 × 10^−6^ (Table S1). Thus, the minimum concentration of amphiphiles for the formation of micelles was remarkably low. This may be beneficial with regard to formulation stability, especially after dilution prior to or after intravenous application. As a result, CMC was determined as DL-G1: 2.5 × 10^−6^, DL-G2: 3.7 × 10^−6^, DL-G3: 5.0 × 10^−6^.

### 2.2. Drug Loading Studies

**Figure 2:**
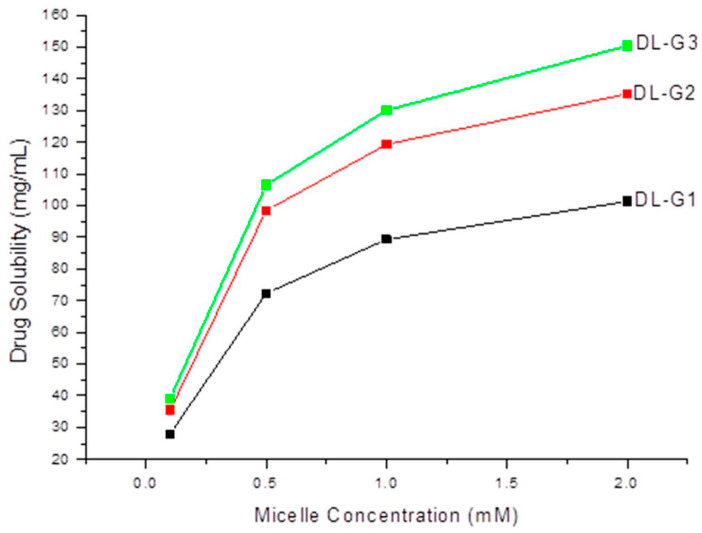
Solubility of 5-FU in different concentration of DL-G1, DL-G2, and DL-G3 micelles.

The effect of micelle concentration on the solubility of 5-FU was measured at 37 °C and the results are shown in the Figure 2. It was observed that the solubility of 5-FU was approximately 2.8 to 15 times better than that of water. For higher generations of dendrimer, the solubility of 5-FU was increased, as expected, but with the increasing concentrations, the solubility increase was less than expected. This might have been due to newly forming micelles with increases in concentration. This might also have been due to the solubilization process itself, which is a very slow distribution process. Nevertheless, a high solubilization of the poor water-soluble drug was achieved with this method, in the absence of any organic solvent and heat.

### 2.3. Cytotoxic Activity Results

Potential anti-proliferative activity of PAMAM-conjugated di-n-dodecylamine micelles (DL-G1, DL-G2, DL-G3) on AGS cells were evaluated by WST-1 assay. Initially, DL-G1, DL-G2, and DL-G3 were used to determine whether treatment of AGS cells with micelles without 5-FU drug loading could result in any cytotoxicity. Therefore, the cells were exposed to indicated concentrations of DL-G1, DL-G2, DL-G3 (10, 25, 50, 100, 250, and 500 µM) for 24 h, and the relative cell viability (%) was calculated.

**Figure 3:**
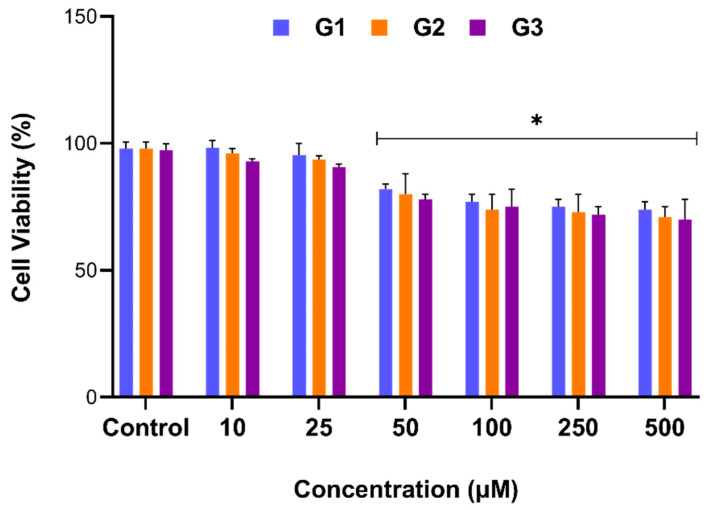
Determination of cytotoxicity of PAMAM-conjugated di-n-dodecylamine micelles (DL-G1, DL-G2, DL-G3). AGS cells were treated with DL-G1, DL-G2, and DL-G3 micelles alone (without 5FU) for 24 h, and cell viability was assessed by WST-1 assay. Data were presented as the percentage of cell viability relative to the control group of each generation. Error bars represent the standard deviation of the mean (* *p* < 0.05).

According to the results, cell proliferation was decreased in a concentration-dependent manner in all generations. While cells at 10 and 25 µM concentrations were near to the control, micelles at higher concentrations reduced cell proliferation. The data revealed that 50 µM and higher concentrations were significantly cytotoxic in all generations (*p* < 0.05, Figure 3). It is known that the toxicity of dendrimers is mainly related to the end groups. Generally, amine-terminated PAMAM dendrimers display concentration-dependent toxicity, which increases with generations [8,11,23]. As the treatment with micelles at elevated doses reduced the cell proliferation, the 25 µM concentration, which has a greater drug-loading capacity than 10 µM (as there is no significant difference in toxicity between 10 and 25 µM) was used in further experiments during the study.

**Figure 4:**
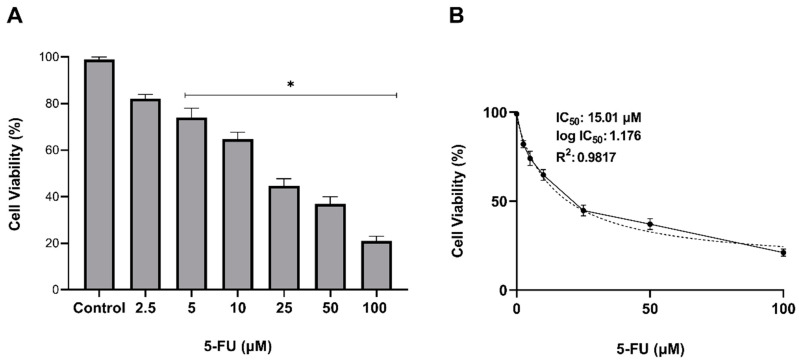
Determination of the antiproliferative effect of 5-FU on AGS cells. To assess the IC50 of 5-FU, cells were exposed to 2.5, 5, 10, 25, 50, and 100 µM 5FU for 24 h, and cell viability was evaluated by WST-1 assay. The percentage of cell viability relative to untreated (control) cells (**A**) and IC50 value of 5-FU (**B**) were presented in the graphs. Error bars represent the standard deviation of the mean (* *p* < 0.05).

In order to assess the IC50 of 5-FU on AGS cells, elevating concentrations in the range of 1 to 100 µM 5-FU were applied to the cells, and the inhibition of cell proliferation was measured for 24 h using the WST-1 assay. According to the results, 5-FU showed significantly toxic effects in all concentrations upon its administration on AGS cells. The IC50 value of 5-FU for AGS cells was determined to be 15 µM, exhibiting 50.1 ± 1.7% cell death in 24 h (*p* < 0.05; Figure 4). Therefore, micelles were loaded with 15 µM 5-FU for further experiments.

AGS cells were exposed to 15 µM of 5-FU-loaded G1, G2, and G3 di-n-dodecylamine micelles for 24 h, and the percentage of cell viability was assessed by WST-1 assay.

Treatment with the drug-loaded form of micelles exhibited remarkably more cytotoxicity than treatment with the drug itself. While 15 µM 5-FU induced 50.1 ± 1.7% cell death, 5-FU-loaded DL-G1 caused higher toxicity, with 59.0 ± 6.5% cell death in 24 h.

Micelles are composed of lipids similar to those present in biological membranes and, therefore, they are readily accepted into the cells via endocytosis [16]. Our results suggest that the micelles facilitate the entry of the drug into the cell; thus, higher toxicity was observed in the drug-loaded micelles.

**Figure 5:**
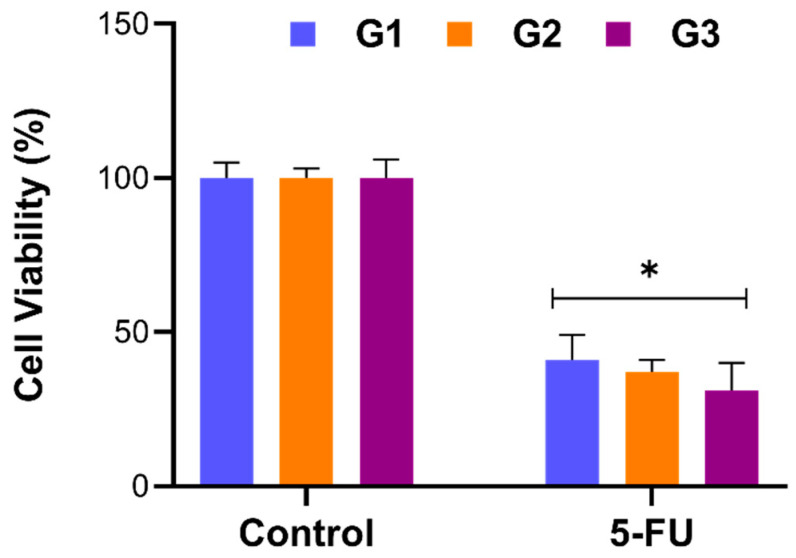
Evaluation of the cytotoxic activity of 5FU-loaded PAMAM-conjugated di-n-dodecylamine micelles (DL-G1, DL-G2, DL-G3). AGS cells were exposed to 15 µM 5FU-loaded 25 µM DL-G1, DL-G2, and DL-G3 micelles for 24 h, and cell viability was assessed by WST-1 assay. Data were presented as the percentage of cell viability relative to the control group of each generation. Error bars represent the standard deviation of the mean (* *p* < 0.05).

Moreover, a generation-dependent gradual decline in cell viability was observed among 5-FU-loaded micelles. The 5-FU-loaded G1 and G2 micelles showed significant cytotoxicity on AGS cells, inducing 59.0 ± 6.5% and 63.7 ± 3.2% cell death, compared with control cells(*p* < 0.05, Figure 5). Di-n-dodecylamine micelles in the third and highest generation (G3) significantly inhibited cell proliferation, demonstrating 69.1 ± 7.3% cell death and exhibiting a significant antiproliferative effect, compared with G1 micelles (*p* < 0.05, Figure 5). The interaction between negatively charged cell membranes and the increased positively charged dendrimer surface can explain why increased generations of cationic dendrimers are more cytotoxic. This increased interaction caused the cell membrane to be damaged, causing nanopores to develop, which ultimately caused the cell to die. At the same time, as the generation increased, the drug-carrying capacity of the denrimer increased and caused more cellular death [24].

Taken together, these data suggest that 5-FU loaded G1, G2, and G3 di-n-dodecylamine micelles possess anti-proliferative activity and induce cell death in a generation-dependent manner, and especially that G3 micelles significantly increase cell death, compared with G1.

## 3. Materials and Methods

### 3.1. Chemicals and Reagents

All chemicals and solvents were reagent grade and were used as purchased without further purification. Electronic spectral studies were conducted on a Shimadzu 2850 model UV-VIS spectrophotometer. ATR spectra were recorded as solid or liquid on Bruker Alpha-P in the range of 4000–400 cm^−1^. Routine ^1^H (400 MHz) and ^13^C (100 MHz) spectra were recorded in ^13^CDC at ambient temperature on a Bruker Ultrashield Plus 400 MHz instrument. Chemical shifts (δ) were expressed in units of parts per million relative to TMS. Micelle images were studied on HR-SEM. The analytical data, mass, ATR, NMR, and physical properties were summarized for each experiment.

For in vitro assays, Dulbecco’s Modified Eagle’s Medium (DMEM), fetal bovine serum (FBS), and penicillin-streptomycin were purchased from Thermo Fisher Scientific (MA, USA). The 5-FU and the WST-1 cell proliferation reagent were purchased from Sigma-Aldrich (Munich, Germany).

### 3.2. Synthesis of PAMAM Dendron Bearing Lipids

**Figure 6:**
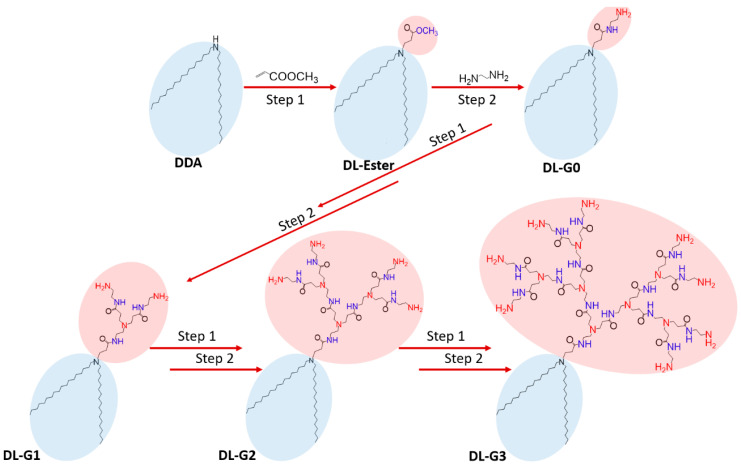
Synthetic Route of PAMAM Dendron-Bearing Lipids.

A series of polyamidoamine dendron-bearing lipids were synthesized by repetition of exhaustive Micheal addition with methyl acrylate using di-n-dodecylamine as the starting material and subsequent exhaustive amidation with ethylenediamine, as reported by Tomalia [25,26,27]. The synthetic route for the polyamidoamine dendron-bearing lipids is shown in Figure 6.

*DL-Ester.* 4 g. Di-n-dodecylamine was dissolved in 25 mL methyl acrylate and the resultant solution were refluxed at 85 °C. After 48 h, methanol and unreacted methyl acrylate were removed under vacuum. As result, white powder of DL-G-0.5 was achieved. ATR (solid, cm^−1^): 2913 ν(C−H), 1736 ν (C=O), 1472, 1441 δ(C−H),1333, 1158 ν(C−O).

*DL-G0.* 3 g. of DL-G-0.5 was dissolved in 80 mL of methanol. The resultant solution was added to 200 mL of ethylenediamine dropwise and the reaction was carried out with constant stirring at 45 °C for 72 h. Methanol and the unreacted ethylenediamine were removed under vacuum. White powder of DL-G0 was obtained. ATR (solid, cm^−1^): 3285, 3201, 3159 ν(N−H), 2910 ν(C−H), 1655 δ(N−H), 1494, 1446 δ(C−H),1314, 1248 ν(N−C).

*DL-0.5*. This was prepared from DL-G0. A total of 2.5 g of DL-G0, dissolved in 36 mL of methanol, was added to 100 mL methy acrylate and the mixed solution was stirred at 35 °C for 72 h. Methanol and the unreacted methy acrylate were removed under vacuum and light yellow lipid was obtained. ATR (liquid, cm^−1^): 2955 ν(C−H),1741ν(C=O), 1481, 1448 δ(C−H).1379, 1178 ν(C-O).

*DL-G1*. This was prepared from DL-G0.5. One g of DL-G0.5, dissolved in 40 mL of methanol, was added to 75 mL of ethylenediamine and the mixed solution was stirred at 45 °C for 72 h. The yellow DL-G1 lipid was obtained after removing excess ethylenediamine and methanol under vacuum. ATR (liquid, cm^−1^): 3282, 3245, 3159 ν(N−H), 2910 ν(C−H), 1638 δ(N−H), 1474, 1423 δ(C−H).1312, 1262 ν(N−C). ^1^H NMR (CDCl_3_), δ_H_ ppm: 1.18 (s, CH_3_(CH_2_) _9_), 1.33 (m, CH_2_CH_2_N), 2.19 (m, CH_2_CONHCH_2_CH_2_NH_2_), 2.77 (t, CH_2_NH_2_), 3.04 (m, CONHCH_2_), 3.40 (m, CH_2_CH_2_NH_2_), 5.11 (m, NH_2_), 7.30, 8.24 (m, CONH). ^13^C NMR (CDCl_3_), δ_C_ ppm: 27.65, 28.32, 29.73 (CH_3_(CH_2_)_10_), 44.88, 46.33, (CH_2_CH_2_NH_2_), 49.45, (CH_2_CH_2_CONHCH_2_CH_2_NH_2_), 54.71, (CONHCH_2_CH_2_), 173.4 (CONH).

*DL-G1.5*. A total of 1.5 g DL-G1, dissolved in 100 mL methanol, was added to 200 mL of methyl acrylate and the mixed solution was stirred at 35 °C for 72 h. Methanol and unreacted methyl acrylate were removed under vacuum. Yellow lipid was obtained. ATR (liquid, cm^−1^): 2988 ν(C−H),1732ν(C=O), 1476, 1451 δ(C−H).1217, 1184 ν(C-O).

*DL-G2.0*. A total of 1.3 g of DL-G1.5, dissolved in 50 mL of methanol, was added to 150 mL ethylenediamine and the mixed solution was stirred at 45 °C for 5 days. Methanol and unreacted ethylenediamine were removed under vacuum. Deep-yellow lipid was obtained. ATR (liquid, cm^−1^): 3342, 3277, 3198 ν(N−H), 2926 ν(C−H), 1640, 1553 δ(N−H), 1460, 1443, δ(C−H).1280, 1020 ν(N−C). ^1^H NMR (CDCl_3_), δ_H_ ppm: 1.20 (s, CH_3_(CH_2_)_9_), 2.45 (m, CH_2_CONHCH_2_CH_2_NH_2_), 2.76, 2.81 (t, CH_2_NH_2_), 3.27 (m, CONHCH_2_), 3.46 (m, CH_2_CH_2_NH_2_), 5.15 (m, NH_2_), 7.29, 8.61 (m, CONH). ^13^C NMR (CDCl_3_), δ_C_ ppm: 41.51, 44.86, (CH_2_CH_2_NH_2_), 50.60, (CH_2_CH_2_CONHCH_2_CH_2_NH_2_), 52.02, (CONHCH_2_CH_2_), 177.5 (CONH).

*DL-G2.5*. A total of 1.5 g of DL-G2, dissolved in 100 mL of methanol, was added to 100 methy acrylate and the mixed solution was stirred at 35 °C for 5 days. Methanol and the unreacted methyl acrylate were removed under vacuum. Deep-yellow lipid was obtained. ATR (liquid, cm^−1^): 2991 ν(C−H),1752ν(C=O), 1474, 1452 δ(C−H).1327, 1192 ν(C-O).

*DL-G3*. One g of DL-G2.5, dissolved in 50 mL of methanol, was added to 75 mL of ethylenediamine and the mixed solution was stirred at 45 °C for 1 week. The remaining methanol and ethylenediamine were removed under vacuum. Dense, deep-yellow lipid was obtained. ATR (liquid, cm^−1^): 3330, 3272, 3183 ν(N−H), 2927 ν(C−H), 1638 δ(N−H), 1461, 1438 δ(C−H).1279 ν(N−C). ^1^H NMR (CDCl_3_), δ_H_ ppm: 1.21, 1.25 (br, s, CH_3_(CH_2_)_9_), 2.35, 2.37 (br, m, CH_2_CONHCH_2_CH_2_NH_2_), 2.62, 2.68 (m, CONHCH_2_ CH_2_) 2.75 (t, CH_2_CH_2_CONHCH_2_CH_2_), 2.82 (m, CH_2_NH_2_)3.29 (m, CONHCH_2_), 3.45 (m, CH_2_CH_2_NH_2_), 5.21 (m, NH_2_), 7.29, 8.65 (m, CONH). ^13^C NMR (CDCl_3_), δ_C_ ppm: 27.65, 28.32, 29.73 (CH_3_(CH_2_)_10_), 44.88, 46.33, (CH_2_CH_2_NH_2_), 49.45, (CH_2_CH_2_CONHCH_2_CH_2_NH_2_), 54.71, (CONHCH_2_CH_2_), 175.8 (CONH).

### 3.3. Preparation of PAMAM Dendron Lipid Containing Liposomes

Micelles were made by direct dissolution of amphiphilic DL-G1, DL-G2, and DL-G3 dendron lipids into PBS (pH = 7.4) at 50 °C for 5 min. in 0.1, 0.5, 1, and 2 mM concentrations. The icelle size and the poldispersity index (PDI) were determined by a scanning electron microscope (SEM). The samples for the SEM were prepared by dropping a few drops of the micelle solution on a thin layer and desiccating them in a desiccator.

### 3.4. Characterisation of Liposomes

The 5-FU was loaded into micelles by the excess method. One mL of DL-G1, DL-G2, and DL-G3 solutions in 0.1, 0.5, 1, and 2 mM concentrations were put into Eppendorf tubes; then, 100 mg of 5-FU was added to each. The tubes were put into a shaker for 3 h at 37 °C. After that, 100 mg more 5-FU was added to the ones that were fully or nearly dissolved and put back into the shaker for 24 h at 37 °C. The undissolved drug and the micelle solutions were separated by using syringe filters with 0.24 μm pore size [6]. The drug content was determined by a UV-Vis spectrometer, by analyzing the peaks at 265 nm.

### 3.5. Determination of Critical Micelle Concentration (CMC)

In order to determine the CMC, UV-Vis spectrophotometry was used. The micelles were determined at 221 nm wavelength (Figure S1). Starting from 1 mM, the micelle solution was diluted until the peak disappeared. As CMC defines the concentration at which micelles are formed, it is also equal to the concentration at which micelles disappear [28].

### 3.6. In Vitro Assays

#### 3.6.1. Cell Culture

AGS (CRL-1739) cells were purchased from the American Type Culture Collection (ATCC) company, thawed, passaged, and stored in liquid nitrogen for further experiments. The cells were subcultured in DMEM containing 10% FBS and 0.1 mg/mL penicillin-streptomycin and incubated in a humidified atmosphere of 5% CO_2_ at 37 °C. The medium was refreshed every 48 h and the cells were passaged upon 70–80% confluency for continuous culture.

#### 3.6.2. Cytotoxicity Assay

The cytotoxic activity of 5-FU- and PAMAM-conjugated di-n-dodecylamine (G1, G2, G3) micelles on AGS cells with and without 5-FU was tested using the WST-1 cell proliferation assay. WST-1 was used to evaluate cell viability according to mitochondrial dehydrogenase activity, which cleaves the tetrazolium salt WST-1 into formazan dye. The WST-1 assay was performed according to the manufacturer’s instructions. The cells were seeded into triplicate 96 well plates at a density of 1.5 × l0^4^ cells/well and incubated overnight. Initially, to determine the IC50 value of 5-FU, AGS cells were exposed to various concentrations of 5-FU (1, 2.5, 5, 10, 25, 50 µM) for 24 h. Second, to evaluate the cytotoxicity of the micelles themselves, the cells were treated with various concentrations of G1, G2, and G3 micelles (10, 25, 50, 100, 250, and 500 µM) for 24 h. Finally, to analyze the anti-proliferative activity of 5-FU-loaded micelles in all generations, the cells were treated with 15 mM 5-FU-loaded 25 µM DL-G1, DL-G2, and DL-G3 micelles for 24 h. After incubation, WST-1 solution was added to the cells and incubated for 4 h. Finally, absorbance was read at 450 nm using a microplate reader (Synergy H1, Biotek).

#### 3.6.3. Statistical Analyses

Data were evaluated by a one-way ANOVA test and an unpaired Student’s *t*-test using the SPSS 25 program. All of the experiments were performed in triplicates and the results were presented as mean ± standard deviation (SD). *p* values less than 0.05 (* *p* < 0.05) were considered as statistically significant.

## 4. Conclusions

The anticancer drug 5-FU has a restricted bioavailability because of its lower solubility and low bioavailability In this work, PAMAM bearing dendron lipids with three different generations have been discussed as for drug-delivery application. It was observed from the phase solubility studies that the solubility of 5-FU increased proportionally with the increasing amount of added dendrimer concentrations. Also, the results showed that their ability to encapsulate these drugs increased with increasing dendrimer generation. Moreover, 5FU-loaded PAMAM conjugated DL micelles significantly inhibit cell proliferation of AGS cells and exhibited cytotoxic activity which increases with generation. The utilization of drug-loaded dendritic micelles is a promising approach to overcome the high systemic toxicity and low solubility of conventional chemotherapy drugs. Further studies are encouraged to evaluate the role of DL micelles as a drug vehicle in various types of tumors in animal models.

## Data Availability

Not applicable.

## Supplementary Materials

The following supporting information can be downloaded at: https://www.mdpi.com/article/10.3390/molecules27227817/s1. Figure S1: Analysis of 5-FU (λ_5-FU_ = 265 nm) and micelle (λ_micelle_ = 221 nm) in ethanol by Uv-Vis spectroscopy; Figure S2. FT-IR analysis of DL-G-0.5; Figure S3. FT-IR analysis of DL-G0; Figure S4. FT-IR analysis of DL-G0.5; Figure S5. FT-IR analysis of DL-G1; Figure S6. FT-IR analysis of DL-G1.5; Figure S7. FT-IR analysis of DL-G2; Figure S8. FT-IR analysis of DL-G2.5; Figure S9 FT-IR analysis of DL-G3; Figure S10: 1H NMR analysis of A) DL-G1 B) DL-G2 C) DL-G3; Figure S11: ^13^C NMR analysis of A) DL-G1 B) DL-G2 C) DL-G3; Table S1: CMC and particle characteristics of micellar dispersions.
