# Supplementary material for: Polyamidoamine Dendron-Bearing Lipids as Drug-Delivery Excipients
Source: Molecules. 2022 Nov 13;27(22):7817. doi: 10.3390/molecules27227817 (PMC9697672; doi:10.3390/molecules27227817)
Supplement: Supplementary file 1 [file molecules-27-07817-s001.zip › molecules-2009962-supplementary.pdf]

-Suplementary Informations-

Polyamidoamine Dendron-Bearing Lipids as Drug-Delivery Excipients

Ender Sarigul [1], Merve Zaim [2], Mehmet Senel [3,*], Tugba Sagir [4] and Sevim Isik [5,*]

[1] BSI Group, Kozyatagi, Istanbul 34742, Turkey;
[2] SANKARA Brain and Biotechnology Research Center, Avcilar, Istanbul 34320, Turkey;
[3] Department of Biochemistry, Faculty of Pharmacy, Biruni University, Istanbul 34010, Turkey
[4] Pim Grup Cosmetics Consultancy, Gokturk, Istanbul, 34077, Turkey;
[5] Department of Molecular Biology and Genetics, Faculty of Science and Engineering, Uskudar University, Uskudar, Istanbul 34662, Turkey
* Correspondence: msenel81@gmail.com or msenel@biruni.edu.tr (M.S.); sebimusan@gmail.com or sevim.isik@uskudar.edu.tr (S.I.)

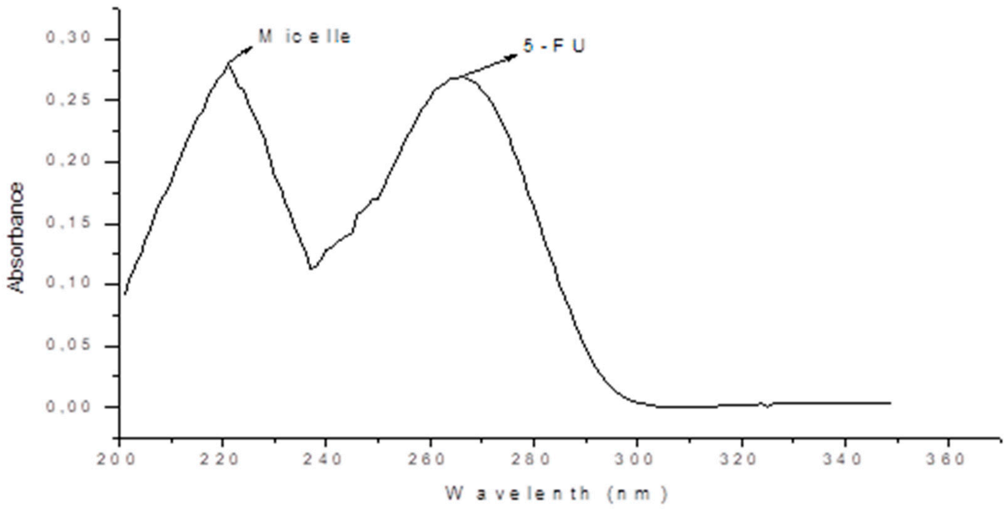

Figure S1. Analysis of 5-FU ($\lambda_{5\text{-}FU}$ = 265 nm) and micelle ($\lambda_{micelle}$ = 221 nm) in ethanol by Uv-Vis spectroscopy.

Table S1

CMC and particle characteristics of micellar dispersions.

DL-	CMC (in mM)	c = 2 mM	
		d (nm)	PDI
G1	2.5×10^{-6}	8.6	0.15
G2	3.7×10^{-6}	11.5	0.18
G3	5.0×10^{-6}	13.8	0.20

FT-IR Analysis of DL-G-0.5

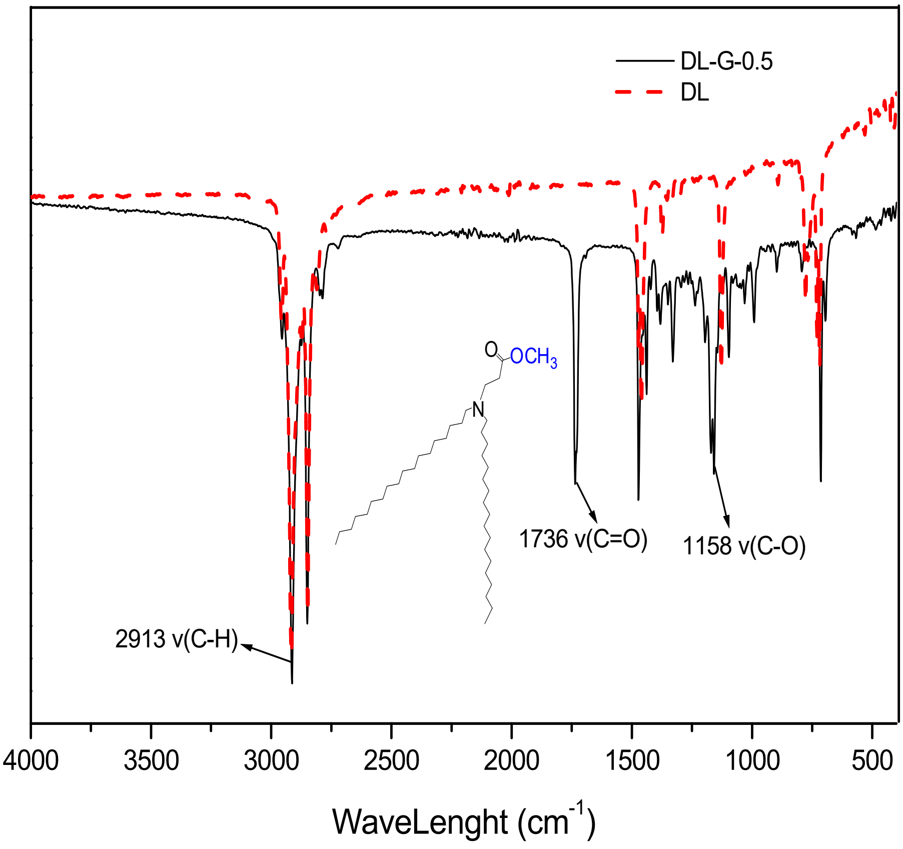

Figure S2. FT-IR analysis of DL-G-0.5

First reaction was made between di-n-dodecylamine and methyl acrylate and the resulting material have characteristic C=O and C-O groups and those groups can be seen on the FT-IR spectra above.

FT-IR Analysis of DL-G0

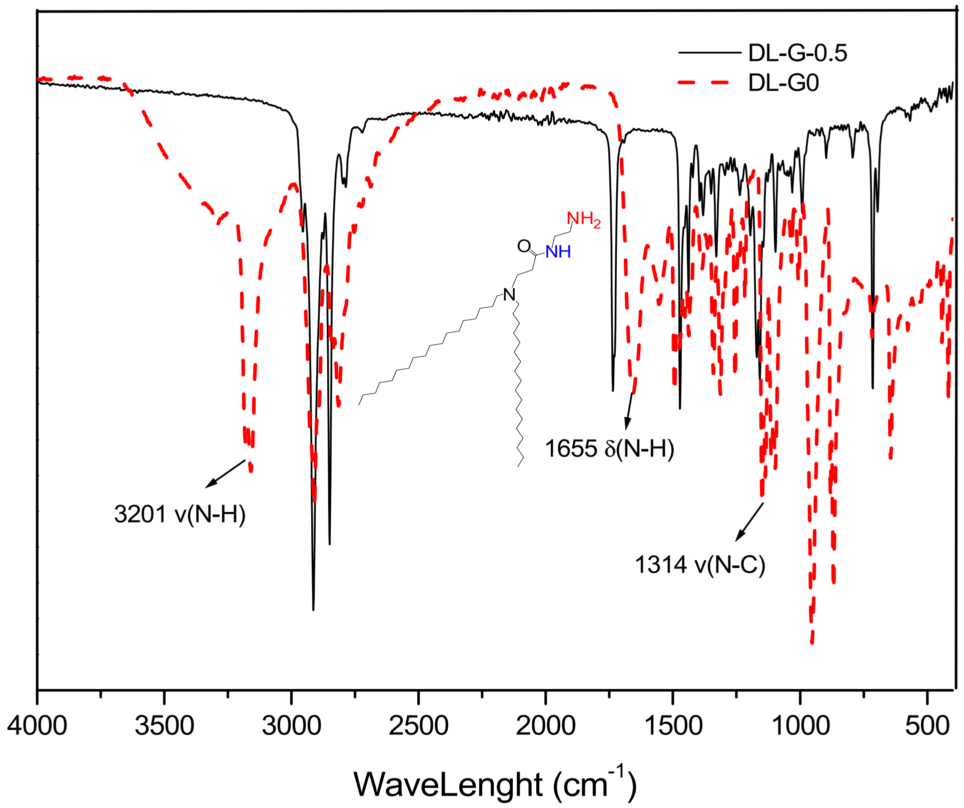

Figure S3. FT-IR analysis of DL-G0

After the second reaction, zero generation (DL-G0) was acquired. As can be seen from the structure of DL-G0, the characteristic groups for FT-IR were N-H and N-C. N-H can be both seen as stretching and bending peaks while N-C stretching peak can also be seen. As only side groups can give strong peaks in a bulky materials like dendrimers, C=O and C-O can no longer be detected.

FT-IR Analysis of DL-G0.5

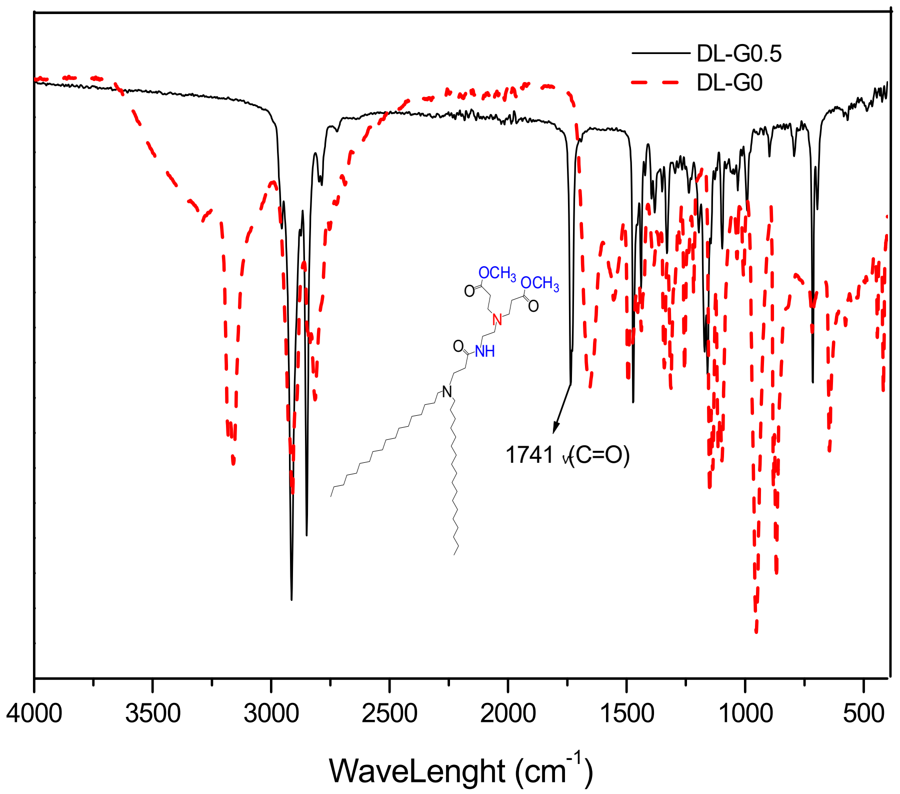

Figure S4. FT-IR analysis of DL-G0.5

After the third reaction, DL-G0.5 was acquired. As can be seen from the above spectra N-H and N-C peaks are gone and C=O peak is occurred after the reaction. So, the reaction is most probably occurred as we intended.

FT-IR Analysis of DL-G1

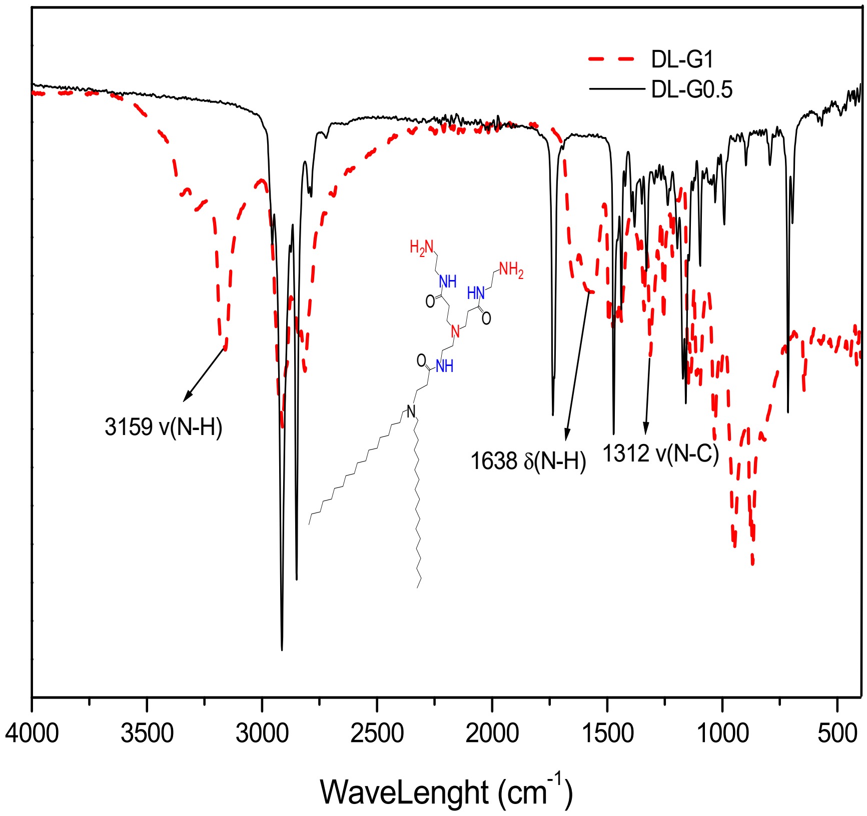

Figure S5. FT-IR analysis of DL-G1

After the fourth reaction DL-G1 was acquired. As can be seen from above one of the end group of this material is amine. At the FT-IR spectra of this material N-H and N-C peaks are seen as expected.

FT-IR Analysis of DL-G1.5

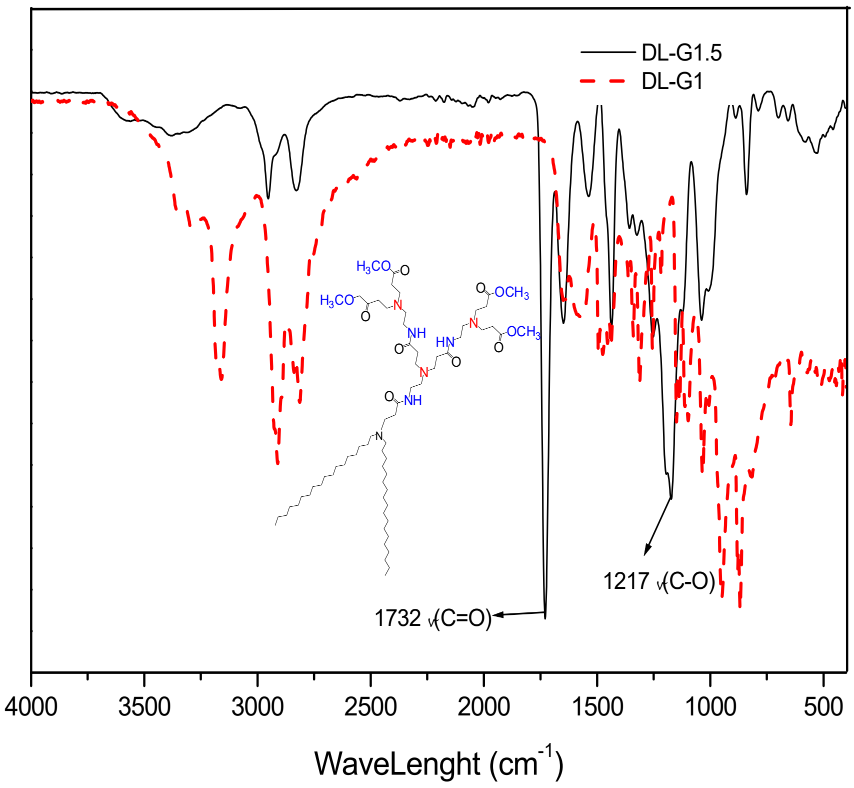

Figure S6. FT-IR analysis of DL-G1.5

After the fifth reaction DL-G1.5 was acquired. As can be seen from above, one of the end group of this material is ester. At the FT-IR spectra of this material C=O and C-O peaks are seen as expected.

FT-IR Analysis of DL-G2

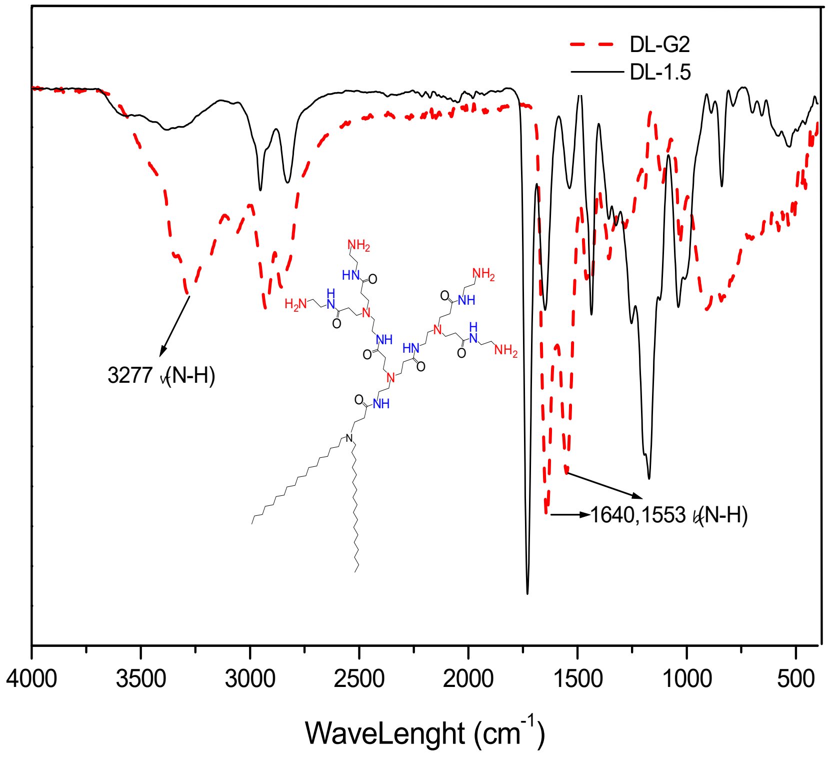

Figure S7. FT-IR analysis of DL-G2

After the sixth reaction DL-G2 was acquired. As can be seen from above, one of the end group of this material is amine. At the FT-IR spectra of this material N-H and N-C peaks are seen as expected.

FT-IR Analysis of DL-G2.5

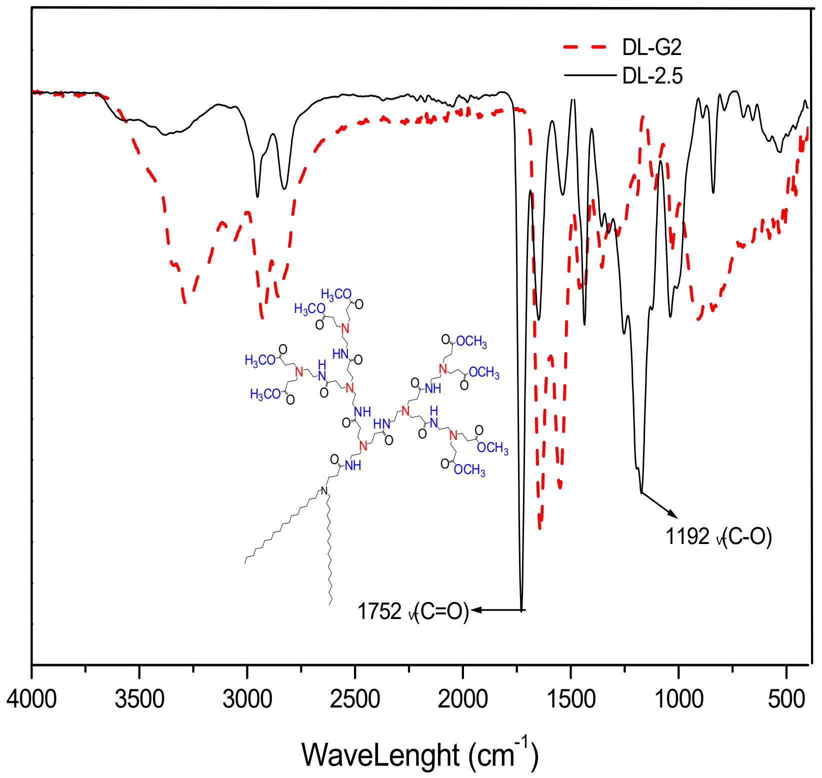

Figure S8. FT-IR analysis of DL-G2.5

After the sixth reaction DL-G2.5 was acquired. As can be seen from above, one of the end group of this material is ester. At the FT-IR spectra of this material C=O and C-O peaks are seen as expected.

FT-IR Analysis of DL-G3

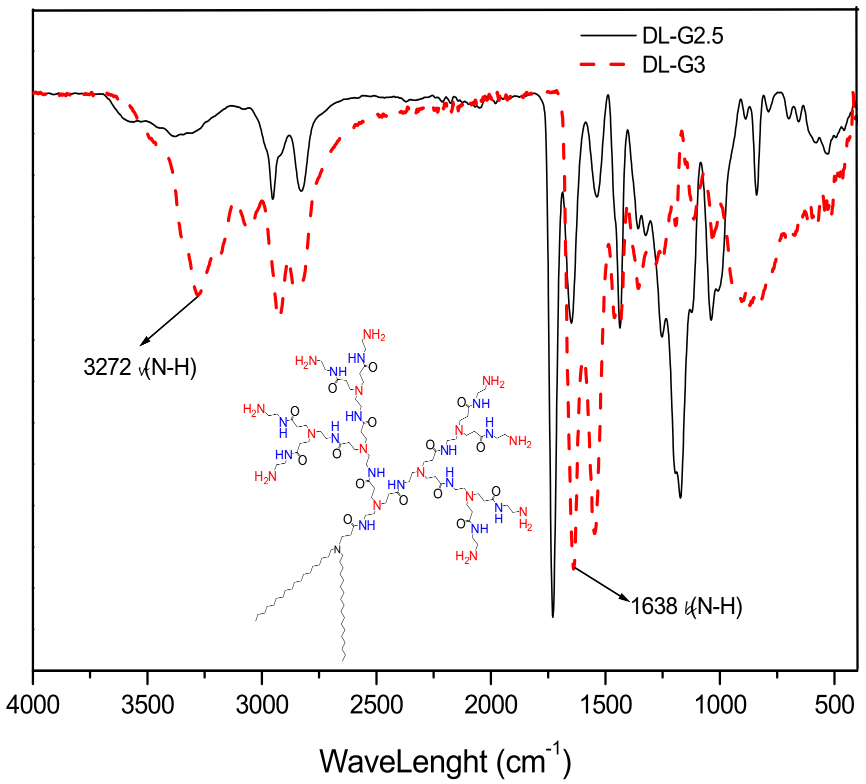

Figure S9 FT-IR analysis of DL-G3

After the seventh and last reaction DL-G3 was acquired. As can be seen from above, one of the end group of this material is amine. At the FT-IR spectra of this material N-H and N-C peaks are seen as expected.

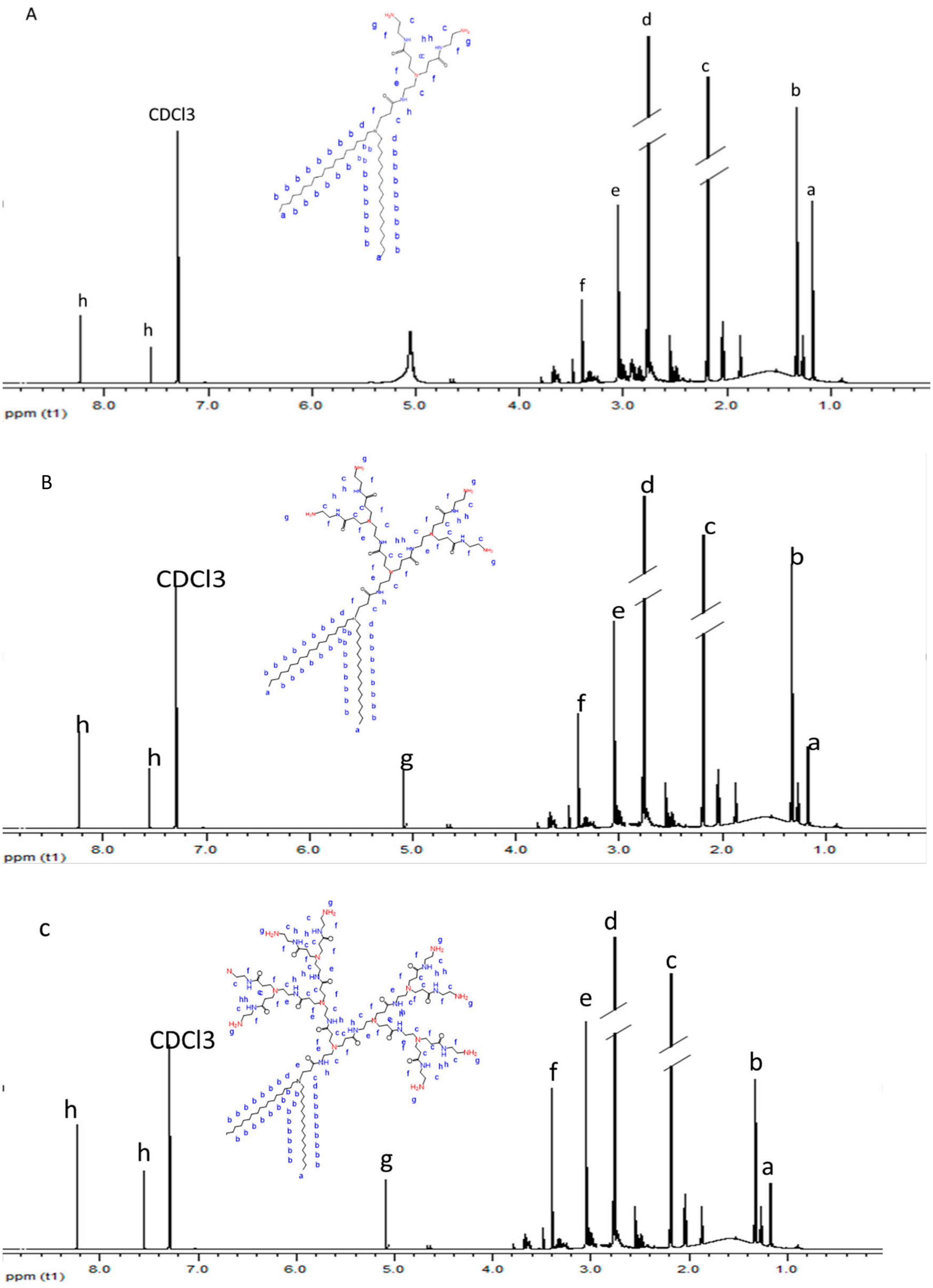

Figure S10: ^{1}H NMR analysis of A) DL-G1 B) DL-G2 C) DL-G3

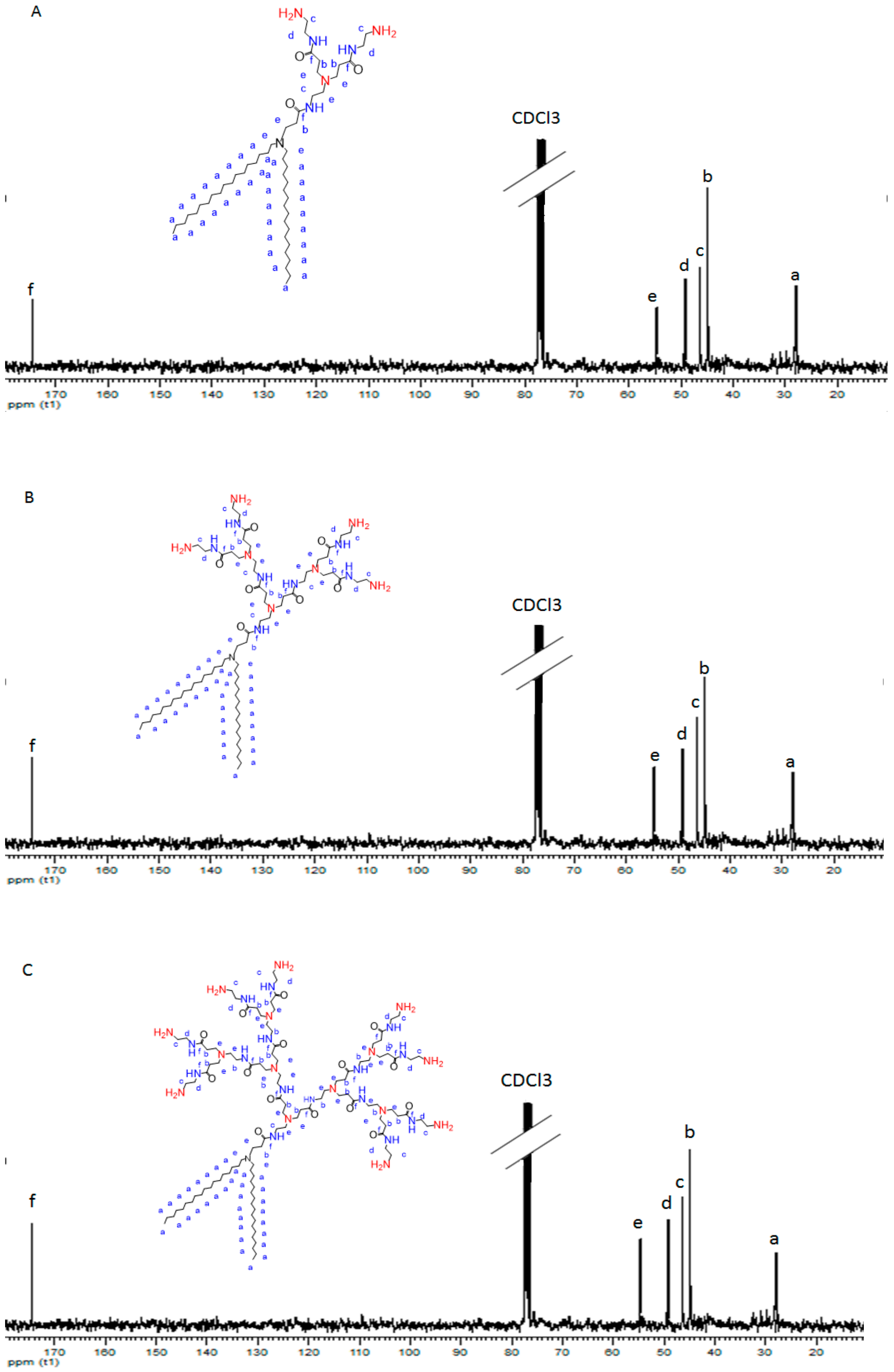

Figure S11: ^{13}C NMR analysis of A) DL-G1 B) DL-G2 C) DL-G3
